# Supplementary material for: Effects of Sugarcane/Peanut Intercropping on Root Exudates and Rhizosphere Soil Nutrient
Source: Plants (Basel). 2024 Nov 20;13(22):3257. doi: 10.3390/plants13223257 (PMC11598586; doi:10.3390/plants13223257)
Supplement: Supplementary file 1 [file plants-13-03257-s001.zip › plants-3135238-supplementary.pdf]

Effects of sugarcane/peanut intercropping on root exudates and rhizosphere soil nutrient

Table S1. Enrichment of metabolic pathways in sugarcane/peanut intercropping

	Metabolic pathway	Hits	Up	Down	P value	Impact	Metabolite name
MP vs IP	Pyrimidine metabolism	3	1	2	0.014	0.094	Uracil; 4,5-Dihydroorotic acid; Dihydrouracil
	Alanine, aspartate and glutamate metabolism	2	1	1	0.020	0.013	Fumaric acid; N-Acetylaspartylglutamate
	Pantothenate and CoA biosynthesis	2	1	1	0.023	0.059	Uracil; Dihydrouracil
	Beta-Alanine metabolism	2	1	1	0.026	0.067	Uracil; Dihydrouracil
	Phenylalanine metabolism	2	1	1	0.080	0.023	Fumaric acid; Phenyllactate
	ABC transporters	3	0	3	0.092	0.022	2'-Deoxyadenosine; Phthalic acid; Methyl beta-D-galactoside
	Oxidative phosphorylation	1	1	0	0.119	0.100	Fumaric acid
	Tyrosine metabolism	2	1	1	0.124	0.065	Fumaric acid; Beta-Tyrosine
	Arginine and proline metabolism	2	0	2	0.124	0.007	Creatinine; Subaphylline
	Citrate cycle (TCA cycle)	1	1	0	0.146	0.028	Fumaric acid
	Limonene and pinene	2	0	2	0.059	0.029	Perillyl alcohol; Limonene-1,2-diol

MP vs IP	degradation						
	Carbon fixation in photosynthetic organisms	1	0	1	0.134	0.026	Fructose 1,6-bisphosphate
	Valine, leucine and isoleucine biosynthesis	1	1	0	0.134	0.047	Ketoleucine
	Sphingolipid metabolism	1	0	1	0.145	0.094	Sphingosine
	Alanine, aspartate and glutamate metabolism	1	0	1	0.161	0.003	N-Acetylaspartylglutamate
	Phenylalanine, tyrosine and tryptophan biosynthesis	1	0	1	0.192	0.008	Fructose 1,6-bisphosphate
	Pentose phosphate pathway	1	1	0	0.197	0.006	Ribose 1,5-bisphosphate
	Valine, leucine and isoleucine degradation	1	1	0	0.232	0.066	Ketoleucine
	Nicotinate and nicotinamide metabolism	1	1	0	0.293	0.009	6-Hydroxynicotinic acid
	Phosphonate and phosphinate metabolism	1	1	0	0.297	0.015	Ribose 1,5-bisphosphate

Hits, the total number of differentiated metabolites in the target metabolic pathway; P value is the P-value of hypergeometric distribution test. The smaller the P-value is, the more significant the detected differential metabolites are on the pathway. The larger the Impact value of the metabolic pathway, the greater the impact of the detected differential metabolites on the target pathway.
